# Culture-independent analysis of liver abscess using nanopore sequencing

**DOI:** 10.1371/journal.pone.0190853

**Published:** 2018-01-09

**Authors:** Liang Gong, Yao-Ting Huang, Chee-Hong Wong, Wen-Cheng Chao, Zong-Yen Wu, Chia-Lin Wei, Po-Yu Liu

**Affiliations:** 1 Genome Technologies, The Jackson Laboratory for Genomic Medicine, Farmington, Connecticut, United States of America; 2 National Chung Cheng University, Department of Computer Science and Information Engineering, Chia-Yi, Taiwan; 3 Department of Medical Research, Taichung Veterans General Hospital, Taichung, Taiwan; 4 Department of Veterinary Medicine, National Chung Hsing University, Taichung, Taiwan; 5 Institute for Systems Genomics, University of Connecticut, Connecticut, United States of America; 6 China Medical University, Taichung, Taiwan; 7 Division of Infectious Diseases, Department of Internal Medicine, Taichung Veterans General Hospital, Taichung, Taiwan; 8 Rong Hsing Research Center for Translational Medicine, National Chung Hsing University, Taichung, Taiwan; Northeastern University, UNITED STATES

## Abstract

The identification of microbial species has depended predominantly upon culture-based techniques. However, the difficulty with which types of organisms are cultured implies that the grown species may be overrepresented by both cultivation and plate counts. In recent years, culture-independent analysis using high-throughput sequencing has been advocated for use as a point-of-care diagnostic tool. Although it offers a rapid and unbiased survey to characterize the pathogens in clinical specimens, its accuracy is reduced by the high level of contamination of human DNA. In this paper, we propose using a culture-independent analysis for a *Klebsiella pneumoniae* clinical strain within a liver abscess using nanopore sequencing. Owing to the highly-contaminated cell population within a liver abscess, we managed to reduce the confounding effects of human DNA through the use of DNase and differential centrifugation. Genomic DNA was sequenced through the use of Nanopore MinION sequencer and analyzed using a suite of bioinformatics approaches. *K*. *pneumoniae* was successfully identified along with antibiotic-resistant genes. Our results indicate that, by integrating real-time nanopore sequencing and bioinformatics software, real-time pathogen identification in a liver abscess can be achieved.

## Introduction

Pyogenic liver abscess (PLA) is one of the most common visceral abscesses, of which Asia is a well-known hotspot for its prevalence. The annual incidence of PLA is estimated to be 3.6 cases per 100,000 individuals in the United States [1] and 17.59 per 100,000 in Taiwan [2]. The condition features high risk complications, particularly amongst patients with an immunocompromised status or those infected with high virulent strains [3]. Currently, the microbiological diagnosis of PLA generally depends upon the isolation of a recognized pathogen from the abscess.

The causative organisms of PLA are highly variable. Of these, *Klebsiella pneumoniae* is an emerging pathogen of PLA found mostly in Asia [4]. Previous studies have revealed a higher risk for patients infected with high-virulent strain of *K*. *pneumoniae* in developing metastatic complications and adverse outcomes [5]. Moreover, the rise in drug-resistant *K*. *pneumoniae* within the community further complicates both the selection of antibiotics and hospital infection control [6, 7]. Conventional microbiology culture-based methods commonly used in current PLA diagnosis require a minimum of two to three days to complete species identification, which is suboptimal in the delivery of effective management and infection control measures. However, the difficulty in which types of organisms are cultured implies that the grown species may be biased and overrepresented through cultivation and plate counts.

Culture-independent analysis using next generation sequencing technology has been advocated as a point-of-care diagnostic tools [8, 9]. Although many studies have shown promising results, the short sequence-read data caused many genome-oriented analyses to be considered impractical [10]. Recently, the Nanopore MinION sequencer, a USB-attached miniature device, has shown promise in the ability to sequence long reads in rapid microbial analysis [11]. This technology can potentially be developed as a rapid, cost effective and field-deployable tool for characterizing both microbial diversity and associated sequence variations in clinical settings. Attempts have been made to detect the virus from blood samples [12] and bacteria found in urine samples [13]. Although it offers a rapid and unbiased survey to characterize the pathogen in clinical specimens, its accuracy is reduced by the high contamination ratio of human DNA, particularly in the liver abscess. Therefore, there remains a critical need for a rapid and precise culture-independent diagnosis of *K*. *pneumoniae* strains in liver abscess [14, 15].

In this paper, we outline the culture-independent analysis of crude liver abscess aspirates through the use of Nanopore MinION and compared the results with the conventional culture-based approach.

## Material and methods

### The case

**Fig 1:**
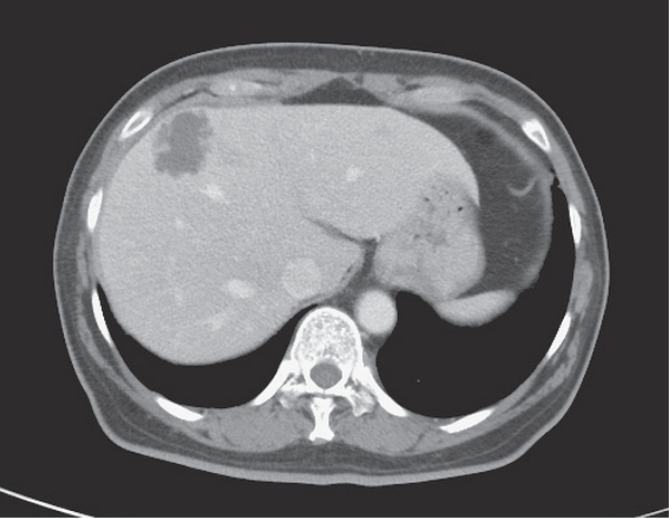
Abdominal CT scan showing a liver abscess.

A 60-year-old woman was presented with a 2-day history of fever and abdominal discomfort, accompanied by poor appetite, nausea, vomiting, and right-upper-quadrant abdominal pain. Her past medical history included type 2 Diabetes mellitus, which had been poorly controlled. Upon admission, her body temperature was 39.2°C, pulse of 132 beats per minute and a blood pressure of 80/52 mm Hg. Tenderness was noted over the right upper quadrant. Laboratory values were: WBC count, 12,550 cells/mm3 (3% bands); albumin, 3.1 g/dL; total bilirubin, 0.6 mg/dL; serum glutamate pyruvate transaminase, 45 IU/dL; and alkaline phosphatase, 154 IU/dL. A CT scan of the abdomen revealed a 3.6 cm septated hypointense area within segment 4 of the liver, indicating an abscess (Fig 1). The patient consented to ultrasound-guided aspiration under ultrasound guidance, with the aspirates sent for both conventional microbiology survey and MinION nanopore sequencing analysis. This research was approved by the Institutional Review Board of Taichung Veterans General Hospital (CE16111B).

### Conventional bacterial strain identification and susceptibility test

Aspirate from the abscess was subjected to culture to order to identify the species. Identification and antimicrobial susceptibility were performed through the Vitek 2 system (bioMérieux, Marcy l′Etoile, France). The Sanger sequencing of 16S rRNA amplified by PCR using universal primers 27F(5’-AGAGTTTGATCCTGGCTCAG-3’) and 1492R(5’-TACGGYTACCTTGTTACGACTT3’) was performed to confirm the species identification.

### Sample collection and DNA extraction

The remaining sample from the routine abscess aspirates was stored at -80°C until DNA extraction was performed. Prior to DNA extraction, a 1ml aspirated abscess was diluted in 1ml of 0.9% sodium chloride. To both minimize the confounding effects of human DNA and improve the efficiency of pathogen detection, we attempted to remove the host human DNA through differential centrifugation. Firstly, the contaminated intact human cells with the liver aspirates were cleared using low speed centrifugation (500g for 5 minutes) at 4°C, followed by a second centrifugation at 800g for 5 minutes at 4°C, in order to remove human cell debris and organelles, including the nucleus. The cleared supernatant was collected for subsequent DNA extraction. The DNA extraction was performed using the DNeasy Blood and Tissue Kit (Qiagen, Valencia, CA, USA) as per the manufacturer’s instructions. The DNA was quantified using a NanoDrop 2000c spectrophotometer (Thermo Fisher Scientific Inc.). In total, we obtained 5060 ng of DNA.

### DNase treatment

To further remove the free human chromosomal DNA which pre-existed in the aspirates, DNA was treated with Plasmid-Safe ATP-Dependent DNase (Epicentre, E3101K) to remove linear genomic DNA from the residual human cells in the abscess aspirates, while maintaining the circular prokaryotic genomic DNA. We prepared the reaction as follows: 42 μL DNA in nuclease free water, 2 μL 25 mM ATP, 5 μL 10X Reaction buffer and 1 μL Plasmid-Safe DNase (10 U). The reaction was incubated at 37°C for 1 hour, followed by heat inactivation using the Plasmid-Safe DNase at 70°C for 30 minutes. A Qubit dsDNA HS Assay Kit (Invitrogen, Q32851) was used to quantify the post-exonuclease treated DNA. We began with 1,990 ng and 2,300 ng DNA, respectively. After the first round, there were 884 ng and 453 ng, respectively. Finally, 106 ng and 86 ng DNA were eluted, respectively.

We next purified the DNA with AMPure XP beads (Beckman Coulter, A63881) by adding 90 μL (1.8X) of resuspended AMPure XP beads to the reaction at room temperature, before mixing well on a rotator for 5 minutes. The mixture was separated on a magnet and the supernatant was discarded. We then kept the beads on the magnet and washed them twice using 200 μL of 70% ethanol, without disturbing the pellet. The DNA was suspended in 22 μL of nuclease-free water.

### Library preparation

To ensure consistency, we prepared two replicates (WIPR002 and WIPR004) using 100 ng and 82 ng of DNA, respectively. Low input genomic DNA libraries were prepared according to the standard protocol using version six of the library preparation kits (ONT, SQK-MAP006 and EXP-LWI001) as supplied by Oxford Nanopore Technologies (ONT). The library was constructed follows:

1. Fragmentation. DNA treated with Plasmid-Safe ATP-Dependent DNase (Epicentre, E3101K) was sheared in a Covaris g-Tube (Covaris, 520079) using a centrifuge (Eppendorf, 5424R) at a speed of 6,000 rpm for 1 minute. The tube was then inverted and the shearing step repeated to collect the fragmented DNA.
2. End-repair and dA-tailing. The fragmented DNA in 50 μL nuclease-free water was incubated in a 7 μL Ultra II End-Prep buffer and a 3 μL Ultra II End-Prep enzyme mix from NEBNext Ultra^™^ II End Repair/dA-Tailing Module (New England Biolabs, E7546S) at a temperature of 20°C for 5 minutes and 65°C for 5 minutes, respectively. The DNA was then purified with AMPure XP beads (Beckman Coulter, A63881). 60 μL (1X) of resuspended AMPure XP beads were added and mixed thoroughly on a rotator for 5 minutes. The beads were then separated on a magnet to remove the supernatant. We kept the beads on the magnet and washed them twice using 200 μL of 70% ethanol without disturbing the pellet followed by re-suspending the pellet in 10 μL of nuclease-free water.
3. Ligation. We prepared the ligation reaction follows: 10 μL of water (DNA), 3.5 μL of Adapter Mix (AMX), 1.5 μL of Hairpin Adapter (HPA) (ONT, SQK-MAP006) and 15 μL of Blunt/TA Ligase Master Mix (New England Biolabs, M0367S). We mixed all the above reagents and incubated them at room temperature for 15 minutes. 1 μL of Low-input Hairpin Tether (LIT) (ONT, EXP-LWI001) was added to the reaction and incubated at room temperature for 15 minutes.
4. Purification. The MyOne C1 beads (Thermo Fisher, 65001) were resuspended at room temperature through a vortex and washed twice with 30 μL of Bead Binding Buffer (BBB) (ONT, SQK-MAP006). 30 μL of washed MyOne C1 beads in BBB were added to the ligated DNA reaction and incubated on a rotator at room temperature for 15 minutes. We then washed the beads twice with 80 μL of BBB and resuspended the pelleted beads in a mix of 10 μL nuclease-free water and 11 μL of Elution Buffer (ELB) (ONT, SQK-MAP006). This is the adaptor-ligated library WIPR002/WIPR004, which is called Pre-sequencing Mix (PSM) in Nanopore sequencing.

### MinION sequencing

The library WIPR002 was sequenced on MinION Mk1 (ONT, 17696) using the R7.3 flow cell (ONT, FLO-MAP103, FAD11732). MinKNOW 0.51.3.40 was used to control the MinION device following the standard 48 hour run scripts. The library WIPR004 was sequenced on MinION Mk1 (ONT, 17690) using the R7.3 flow cell (ONT, FLO-MAP103, FAA10025). MinKNOW 0.51.3.55 was used to control the MinION device following a 24 hour run script (remux every 8 hours). We used the application, What’s In My Pot (WIMP) [11] (ONT, WIMP Bacteria Virus Fungi k24 for SQK-MAP006, version 1.0.4) to perform base calling, bacteria sequence identification, along with sub-species and strain classification. We performed the entire sequencing and WIMP analysis on a single laptop (Apple, MacBook Pro).

### Bioinformatics analysis

Microbial identification and classification were carried out using WIMP, which performs real-time taxonomic classification according to the NCBI taxonomy [11]. The Kraken based [16] WIMP application use pre-built data structure to map all k-mers and make phylogenetic classification and identification of microorganisms. During identification process, the fraction of k-mers from the sequence mapped to the least common ancestor is calculated and represents classification score.

To characterize the identify of all the reads generated by the Nanopore sequencing in addition to those hit in the WIMP database, we performed exhaustive searches using two different aligners; BLAST [17] and minimap2 [18]. The choice of the aligners selected was to ensure that we could achieve sensitivity (BLAST), while optimizing for reads with a higher error rate (minimap2). ONT reads aligned onto the reference genomes of *K*. *pneumoniae* and human hg19 (with an e value 10^−5^) were output for further analysis. Antibiotic-resistant genes were annotated by using a Blastx search of protein-coding genes on the *K*. *pneumoniae* genome against the Comprehensive Antibiotic Resistance Database (CARD) [19].

## Results

### Conventional bacterial strain identification and antimicrobial susceptibility testing

*K*. *pneumoniae* was identified by the Vitek 2 automated system (bioMérieux, Marcy l′Etoile, France). The species identification was confirmed through 16S rRNA gene sequencing. The isolate was susceptible to cefazolin, ceftriaxone, cefepime, ampicillin/sulbactam, piperacillin/tazobactam, gentamicin, amikacin, trimethoprim/sulphamethoxazole, ertapenem, imipenem, and ciprofloxacin.

### MinION sequencing results

**Fig 2:**
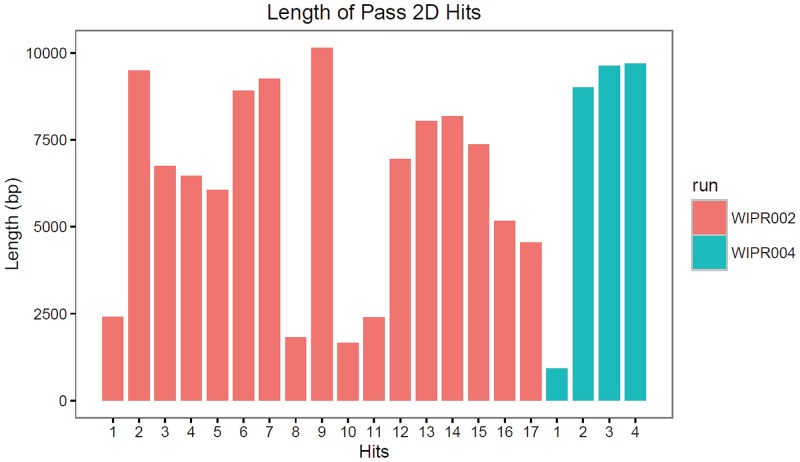
The length distribution of the 2D reads.

The major challenge to analysis of metagenome DNA in clinical PLA aspirates is the host DNA contamination. The host DNA can result from the genomic DNA released from the lysed host tissues or the freely circulating blood cells. Because the relative sizes of nuclear DNA between human and microbial cells are approximately 1,000X (Gb vs. Mb), even a minor degree of host cell contamination will yield a large number of human sequences; resulting in a low efficiency in pathogen identification. To reduce the level of potential host DNA in the specimen, and to improve the sequencing efficiency, we adopted two approaches towards the preparation of the DNA prior to the library construction and sequencing analysis. The aspirate samples were first subjected to low speed centrifugation in order to remove the human cells. The supernatant containing most of the microbial cells was collected and treated with the plasmid-safe DNase to remove residual human DNA in the linear form, while leaving the circular bacterial DNA intact. Two MinION runs were performed with two low input genomic DNA libraries WIPR002 and WIPR004 for 48 and 24 hours, respectively. We observed a high variability in flow cell quality as indicated by the drop in the numbers of active pores after 24 hours. The base calling was achieved with the WIMP application [11] being used simultaneously during the run. In total, we generated 31,696 and 9,395 reads; which accounted for 95 Mb and 40 Mb from WIPR002 and WIPR004, respectively. Among them, 12,028 and 3,835 reads respectively are defined as 2D reads (reads from individual DNA molecules with sequences from both strands). For a summary of the run metrics, see Table 1. For the high-quality pass 2D reads, the mean lengths of WIPR002 and WIPR004 are 4,498 bp and 5,207 bp, respectively, and the maximum lengths are 13,027 bp and 14,359 bp, respectively. The size distribution of the total reads generated matched well with the sequence fragments mapped to the targeted bacterial genome (Fig 2), suggesting a minimal bias in the sample preparation.

**Table 1:** MinION sequencing statistics.

Library Name	Flow cell chemistry	Sequencing time (h)	Active Pores	Total Yield (bp)	Total Reads	Total 2D Yield (bp)	Total 2D Reads	Pass 2D Reads	2D Mean Length	2D Max Length	Pass 2D Hits
WIPR002	R 7.3	48	400	95,209,596	31,696	30,264,362	12,028	1,144	4,498	13,027	17
WIPR004	R 7.3	24	611	40,734,158	9,395	12,985,223	3,835	254	5,207	14,359	4

### Bacteria species identification

For microbial identification and classification, WIMP was adopted to perform real-time taxonomic classification according to the NCBI taxonomy [11]. Using the established WIMP processing pipeline, we successfully identified two subtypes of *K*. *pneumoniae*, NCBI Taxonomy ID 573 and Taxonomy ID 72407 (*K*. *pneumoniae* subsp. pneumoniae) with high confidence. The first strain was supported by 10 reads (7 2D reads and 3 template reads) while the second strain was supported by 11 reads (10 2D reads and 1 template read) in WIPR002 (S1 Table). Similarly, the identical two strains were also identified in WIPR004, but were supported by only four 2D reads (S1 Table). The length distribution for these reads ranged from 1 to 10 Kb in size; suggesting a diverse and non-biased sampling process in the sequencing (Fig 2).

**Fig 3:**
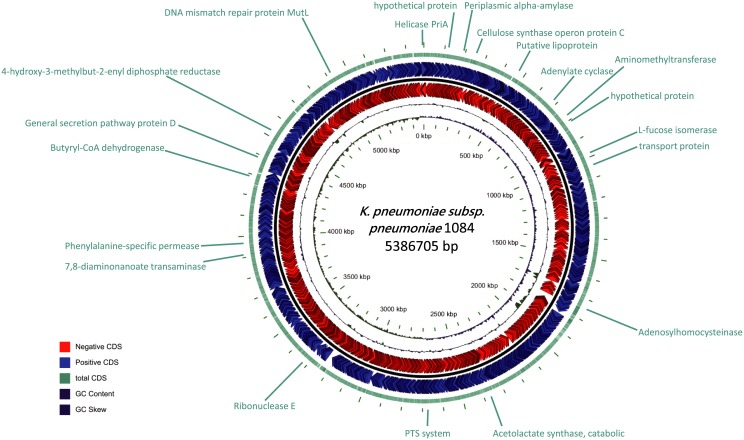
Circular map of the 2D reads in the context of a reference genome and corresponding annotation.

Due to the fact that 2D reads display a higher accuracy, the twenty-one 2D reads were used to perform BLAST analysis. Their results showed that twenty reads originated from the *K*. *pneumoniae* genome, while one read was from plasmid (S2 Table). The twenty genomic reads were randomly distributed across the 5 Mb genome, and covered 120 protein-coding genes found through NCBI gene annotation (Fig 3). Each read covered between one to thirteen genes (with an average ~5 genes). Among the 130 genes sequenced and revealed by ONT reads, seven were annotated as antibiotic-resistant (AR) genes as determined by the Comprehensive Antibiotic Resistance Database (CARD). These seven AR genes may form antibiotic efflux complex or confer resistance to either fluoroquinolones or aminocoumarin antibiotics, which is commonly observed in other strains of *K*. *pneumoniae* (S3 Table).

Because only limited reads can be assigned to the *K*. *pneumoniae* genome, we sought to determine the degree in which there was human host DNA contamination. The total reads generated from each of the two Nanopore datasets were aligned to the human reference genome (hg19) through BLAST analysis, where the % of the reads assigned to human DNA were determined to be 70% and 75% (S4 Table). This data implicated that despite the moderate levels of pathogen DNA enrichment from the iterative purification processes, the majority of the DNA isolated from the aspirates was still of host origin. Outside of the identified *K*. *pneumoniae*, the remaining >20K non-human reads did not share any similarities with any known bacterial species, suggesting the existence of many new bacteria species in the abscess aspirates. The identity of these species can be further characterized through additional nanopore sequence data study.

## Discussion

In our study, culture-independent analysis successfully identified *Klebsiella pneumoniae* along with antibiotic-resistant genes by reducing the confounding effects using DNase and differential centrifugation, as well as a suite of bioinformatics processes. Our results suggest the feasibility of culture-independent analysis using direct sequencing as the molecular diagnostic testing method for rapid pathogen identification.

Prompt detection of pathogens is critical for not only definite treatment, but also surveillance and infection control. Among patients with severe infection, delay in pathogen identification leads to untimely and improper antimicrobial chemotherapy and ultimately higher mortality rates. Approximately half of *K*. *pneumoniae* bacteremia-related deaths occur within the first 72 hours upon admission [20]. However, it often requires 3–5 days for conventional methodology to reach final microbiology diagnosis [21]. Overall, the entire workflow from DNA extraction to strain identification should take approximately 3–4 hours, but can be further reduced through more streamlined and optimized protocol. Specifically, it takes 30 minutes for DNA extraction from the clinical specimen followed by 90 minutes of DNase treatment and an additional 60 minutes for nanopore library preparation. Sequencing is then carried out on MinION, using real-time data analysis by the WIMP pipeline to perform bacterial strain identification. Compared to the current standard practice, this approach in principle is two to three-fold faster, allowing for the potential to obtain more detailed subtyping information at the nucleotide-level. Therefore, metagenomic analysis with next generation sequencing technology of clinical specimens has been advocated as a point-of-care diagnostic tool.

The concern has been raised in several trials that high proportions of human cells would interfere with the analysis, thus reducing the sequencing yield. Hence, the application to human cell-rich samples such as abscess aspirates remains limited, which in turn that downplays the role of metagenomics analysis in real world daily practice. We managed to identify the pathogen from human cell-rich samples using the MinION single-molecule nanopore sequencer, through the application of DNase and differential centrifugation to reduce the contamination of human DNA. Although more than 90% of the reads were mapped to the human genome (S4 Table), the numbers of high quality 2D reads in the two runs performed (12,028 and 3,835, respectively) was equivalent to other report [12]. Further efforts to minimize human host DNA will be the future focus of the research community in order to improve efficiency and reduce the cost of this approach.

The *K*. *pneumoniae* Taiwan/China strain, which is associated with hypervirulent capsular serotype K1 and known for its high mortality rate [3, 22], has been precisely identified by MinION from liver abscess aspirate. Clinical manifestation of the *K*. *pneumoniae* Taiwan/China strain could be aggressive, possibly causing invasive liver abscess, endophthalmitis, and central nervous system infections [23]. Therefore, rapid detection of this invasive strain will allow clinicians to initiate timely management towards it, while alleviating any adverse outcomes. It appears that taking these steps could greatly reduce adverse outcome.

Our analyses also predicted the presence of 7 AR genes encoded by the *K*. *pneumoniae* found in our sample, most of which are efflux pump genes. The detected efflux pump genes in our study belong to two important families of chromosomally encoded bacterial efflux pumps, the Major Facilitator Superfamily (MFS) and the ATP Binding Cassette (ABC) superfamily[24]. Although there was no detectable phenotypic resistance to tested antibiotics in the isolated *K*. *pneumoniae*, the presence of these genes indicated the possibility of emerging resistance and subsequent treatment failure during antimicrobial therapy. It has long been recognized that efflux pumps are involved in the development of multidrug resistance during treatment [25, 26]. Moreover, functional analysis further revealed that both MFS and ABC efflux pumps play roles in the adaption and pathogenicity of multiple organisms [27, 28].

## Conclusions

The MinION single-molecule nanopore sequencer may be a valuable, supplemental tool in clinical diagnostic microbiology. To better integrate this technology into the clinical microbiology laboratory, more real-world experience and studies are still required to determine the appropriate workflow, and to also optimize protocols for sample preparation in various clinical contexts.

## Supporting information

S1 Table Real-time taxonomic classification using WIMP processing pipeline. (XLSX)

S2 Table Results of BLAST analysis of 2D reads. (XLSX)

S3 Table Antibiotic-resistant genes determined by the Comprehensive Antibiotic Resistance Database. (XLSX)

S4 Table Sequence analysis of host DNA contamination. (XLSX)
